# The Role of Cancer-Derived Exosomes in Tumorigenicity & Epithelial-to-Mesenchymal Transition

**DOI:** 10.3390/cancers9080105

**Published:** 2017-08-10

**Authors:** Robert H. Blackwell, Kimberly E. Foreman, Gopal N. Gupta

**Affiliations:** 1 Department of Urology, Loyola University Medical Center, 2160 S. First Ave., Maywood, IL 60153, USA; rblackwell@lumc.edu; 2 Cardinal Bernardin Cancer Center, Loyola University Medical Center, 2160 S. First Ave., Maywood, IL 60153, USA; kforema@luc.edu

**Keywords:** epithelial-mesenchymal transition, exosomes, neoplasm metastasis, neovascularization, pathologic, neoplasm invasiveness, intercellular signaling peptides and proteins

## Abstract

Epithelial-to-mesenchymal transition (EMT) is a process by which epithelial cells lose their basement membrane interaction and acquire a more migratory, mesenchymal phenotype. EMT has been implicated in cancer cell progression, as cells transform and increase motility and invasiveness, induce angiogenesis, and metastasize. Exosomes are 30–100 nm membrane-bound vesicles that are formed and excreted by all cell types and released into the extracellular environment. Exosomal contents include DNA, mRNA, miRNA, as well as transmembrane- and membrane-bound proteins derived from their host cell contents. Exosomes are involved in intercellular signaling, both by membrane fusion to recipient cells with deposition of exosomal contents into the cytoplasm and by the binding of recipient cell membrane receptors. Recent work has implicated cancer-derived exosomes as an important mediator of intercellular signaling and EMT, with resultant transformation of cancer cells to a more aggressive phenotype, as well as the tropism of metastatic disease in specific cancer types with the establishment of the pre-metastatic niche.

## 1. Introduction

Epithelial-to-mesenchymal transition (EMT) represents the process in which an epithelial cell, with normal basement membrane interaction, can undergo biochemical changes to lose the epithelial characteristics and develop a more migratory, mesenchymal phenotype [1,2]. While first described as a fundamental element of organ and tissue differentiation, EMT has been shown to have roles in wound healing, tumor initiation, and progression to metastatic disease [3,4,5,6].

Exosomes are membranous vesicles that are released by cells into the extracellular environment. Exosomes measure 30–100 nm in size and carry a variety of content including a lipid bilayer membrane, DNA, RNA, mRNA, miRNA, bioactive lipids (including prostaglandins, fatty acids, and leukotrienes), as well as intracellular and membrane-bound proteins [7,8]. Originally thought to function in disposal of unnecessary or waste cellular contents, exosomes have now shown involvement in cellular communication and cancer progression via target-cell: exosomal interactions as well as deposition of exosomal content into the target-cell cytoplasm [9,10,11]. As such, prior work has demonstrated the significant role exosomes play in cancer biology, promoting growth and survival of tumor cells distant from the primary tumor, tumor-cell apoptosis inhibition, as well as promoting angiogenesis, migration, invasiveness, and tumor cell viability [2,12,13,14,15,16,17]. Cancer-derived exosomes are now believed to be mediators of metastatic disease and contribute to the tropism of metastasis seen in different cancer cell types [18,19].

## 2. Exosomal Isolation

The ability to isolate exosomes has been demonstrated in cell culture media as well as nearly all bodily fluids examined (blood, urine, saliva, breast milk, etc.) [20,21,22]. The current standard for exosomal isolation involves differential centrifugation at increasing speeds [23]. Low speed centrifugation results in the removal of cellular debris, followed by ultracentrifugation at 100,000× *g*-force to ultimately pellet exosomes. Alternatively, kits exist to isolate exosomes that use magnetic coated beads with exosome-specific antibodies. While these kits avoid the time-intensive ultracentrifugation process, they are not intended for the isolation of large quantities of exosomes [20]. Comparison between the techniques with regard to protein, mRNA, and miRNA yields reveals that either technique is applicable for further research or clinical application; however, for protein analysis, serial ultracentrifugation resulted in a higher level of purity [24,25]. Following isolation, size determination with scanning electron microscopy or nanoparticle tracking analysis can be performed to confirm exosome isolation, with an anticipated result of vesicles ranging 30–100 nm in size [26]. In addition to size alone, the International Society for Extracellular Vesicles recommends characterization and quantification of proteins of any exosomal preparation, including those expected to be present or enriched (e.g., transmembrane or lipid-bound extracellular proteins, cytosolic proteins) and those expected to be absent or underrepresented (e.g., intracellular proteins), to properly characterize exosomes and potentially co-isolated extracellular vesicles [27]. Clinically, there are currently two exosome-based, commercially available diagnostic tests that exist for prostate and lung cancer [28].

## 3. Exosomal Signaling/Cargo Delivery

**Figure 1:**
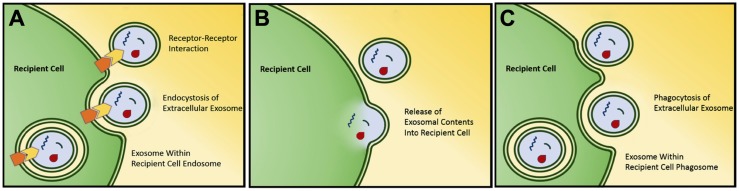
Several mechanisms have been found for interaction and uptake of exosomes by target cells. Most well studied mechanisms are receptor-mediated exosomal uptake (endocytosis) (**A**), direct exosomal fusion with plasma membrane (**B**), and phagocytic exosomal uptake (phagocytosis) (**C**). Used with permission [20].

Exosomes have been found to carry a variety of biomolecules (including protein, DNA, mRNA, miRNA) in their lumen and/or on the surface [29]. Following release, exosomes can then interact with recipient cells to facilitate cell-cell signaling. This interaction comes in the form of direct interaction between exosomal transmembrane or membrane-associated surface proteins with recipient cell receptors resulting in cellular signaling, or by exosomal internalization with subsequent fusion and deposition of exosomal contents [30,31,32] (Figure 1). While not yet demonstrated in vivo, this exosomal internalization has been shown to be at least partially inhibited by treatment with heparin in vitro, as well as non-acidic conditions given alteration in membrane rigidity [32,33].

## 4. Exosomal Proteome Alterations Following Epithelial-to-Mesenchymal Transition

Once mesenchymal transition occurs, there is an alteration in the cancer-derived exosomal proteome. While these changes are not an exact replication of the host cell, these changes affect proteins involved in junction formation, cellular adhesion, communication, and proliferation [34]. Garnier and colleagues investigated these changes to the exosomal proteome in the human squamous cell carcinoma A431 cell line. Comparisons were made between the A431 cell line vs. A431 cells treated with anti-E-cadherin antibody and TGFα to induce a mesenchymal phenotype. Using Ingenuity pathway analysis to characterize the proteome functions, cancer-derived exosomes from the treated cells, compared to untreated cells, demonstrated a marked enrichment in proteins involved in cellular growth and proliferation, cell-to-cell signaling and interaction, cellular movement, and cellular morphology. Integrin signaling, specifically, was involved in 22% of the upregulated proteins, consistent with alterations underlying EMT. This is in contrast to the cellular proteomes of the respective cells lines, which demonstrated minimal if any difference in these functions, suggesting packaging of exosomes occurs in a regulated process. A similar distribution proteome was demonstrated in hypoxia-induced A431 cells, with particular functional clustering in the domains of cellular adhesion and cell-cell junctions [35]. This suggests the exosomal proteome of cancer cells that have undergone mesenchymal transition may reflect their hypoxic nature, or that hypoxia may contribute to EMT in cancer cells [34,36,37].

## 5. Exosomal Stimulation of Angiogenesis

Angiogenesis naturally occurs as a response to pro-angiogenic factors during development and wound healing, but angiogenic pathways may be exploited in the setting of malignancy [38,39]. Gaining access to the host vascular system and establishing a tumor blood supply to supply nutrients, oxygen, and growth factors are rate-limiting with regard to tumor progression, with neovascularization enhancing survival, tumor growth, and dissemination [40,41]. Further, EMT-induced angiogenesis has been shown to be critical in the increase in tumorigenicity of cells undergoing EMT [42].

The role of exosomes in tumor-associated angiogenesis has been well established. In a report by Mineo and coworkers, exosomes isolated from the chronic myeloid leukemia (CML) K562 cell line were shown to induce neovascularization [43]. In vitro, upon application of cancer-derived exosomes to human umbilical endothelial cells (HUVECs), the exosomes stimulated angiotube formation compared to control, with exosomal internalization by the endothelial cells and perinuclear localization during tube formation. An in vivo murine matrigel plug model of angiogenesis demonstrated that cancer-derived exosomes stimulated vascularization to a degree greater than the negative control and equivalent to the positive control. This stimulation of angiogenesis by the cancer-derived exosomes was found to be mediated in a Src-dependent fashion, as dasatinib, a known Src-family kinase inhibitor, blocked the endothelial cell response to cancer exosomes. Further, exosome exposure caused focal adhesion kinase (FAK) phosphorylation, a process that has been shown to be required for EMT [44].

In high-grade bladder cancer, tumor-derived exosomes have similarly been shown to promote angiogenesis as demonstrated by tube formation on the HUVEC assay [14]. This was seen with both cell-line derived and patient-derived bladder cancer exosomal isolations. Beckham and coworkers further elucidated that exosomal EDIL-3, a protein with a known role in angiogenesis and abundantly present in cancer-derived exosomes of both bladder cancer cell lines and bladder cancer patient samples, was necessary to induce tube formation. When cell lines were stably transfected with silencing EDIL-3, exosomal EDIL-3 expression decreased significantly and tube formation was no different than the control. The studies by Mineo and Beckham have regulation of the Src-family of proteins in common, as EDIL-3 is known to activate the FAK-Src-AKT pathway in hepatocellular carcinoma, which may contribute to the explanation for the relationship between cancer-derived exosomes and angiogenesis [45].

Hsu et al. demonstrated similar findings with lung cancer-derived exosomes expressed under hypoxic conditions, which demonstrated enhanced angiogenesis in HUVECs via miRNA delivery [46]. Upon in vitro application of cancer-derived exosomes, increased tube formation was demonstrated compared to HUVECs exposed to normal lung exosomes under both hypoxic and normoxic conditions. In vivo murine matrigel plug models similarly demonstrated increased angiogenesis with cancer-derived exosome application. In this study, *miR-23a*, an miRNA upregulated in hypoxia in lung cancer cells, was identified in the cancer-derived exosomes. *miR-23a* levels were increased in the HUVECs in the in vitro assay following exosome application, and angiogenesis was inhibited in the in vivo model when an *miR-23a* inhibitor was applied concurrent to cancer-derived exosomes. When circulating exosomes were collected from lung cancer patients and healthy control patients’ sera, the cancer patient exosomes demonstrated higher levels of *miR-23a*. When applied in vitro to HUVECs an increase in tube formation was shown. The authors demonstrated that the hypoxia-induced cancer-derived exosomes contained *miR-23a*, which when applied to cells in turn caused the downregulation of PDH1/2 and the resultant upregulation of HIF-1α, an independent promoter of EMT [47].

In renal cell carcinoma, Zhang and colleagues describe the application of cancer-derived exosomes on HUVECs with resultant increased tubularization, as well as an increased HUVEC expression of vascular endothelial growth factor (VEGF) mRNA and protein, a well-known promoter of angiogenesis [48]. Ekström and coworkers, in their work on melanoma-derived exosomes, had similar findings, but they demonstrated that the exosomes carried pro-angiogenic protein VEGF along with IL-6 as intravesical cargo to the epithelial cells [49]. With an application of cancer-derived exosomes to mouse endothelial cell line MS1, tube formation was induced, while exosome-depleted cancer cell culture supernatant did not have any effect. Of note, Ekström described the stimulation of exosome release from melanoma cells in vitro with the application of recombinant-WNT5A, as well as the suppression of exosome-release with the application of WNT5A-silencing RNA. High levels of WNT5A has been implicated in poor prognosis in advanced melanoma patients, and may hypothetically be related to stimulation of exosomal release. This exosome-release effect has been similarly documented with WNT3A treatment of rat microglial cells, and may point to the WNT-family protein regulation of exosome release and signaling [50].

Together, the above studies represent multiple avenues in which cancer-derived exosomes exert pro-angiogenic effects on epithelial cells. Cancer-derived exosomes internalize into endothelial cells to exert this effect, and carry miRNA and protein cargo to affect angiogenesis by a variety of mechanisms. Additional characterization of these effects, in particular the route of signaling (e.g., via receptor-mediated signaling or exosomal uptake and intracellular delivery of exosomal contents) and the pathways involved will elucidate the mechanisms of exosomal-mediated angiogenesis as well as possible therapeutic targets. Further, research is needed to examine the interplay between cancer-derived exosomes, EMT, and angiogenesis to better understand their relationship in promoting tumorigenicity.

## 6. Exosomal Enhancement of Cellular Migration and Invasion

Central to progression of malignancy to advanced disease and metastasis is development of cellular migration and invasion [51,52,53]. Our lab has demonstrated that not only do bladder cancer-derived exosomes increase expression of mesenchymal markers in normal uroepithelial cells, but also alter motility and invasiveness [2]. Following application of muscle-invasive bladder cancer derived exosomes to uroepithelial cells plated on Collagen IV-coated chamber glass, live cell imaging demonstrated increased distance travelled from the origin compared to negative control. Further, in a Transwell system test, bladder cancer-derived exosome application significantly enhanced both migration and invasion of uroepithelial cells.

In prostate cancer, Ramteke and colleagues demonstrated that prostate cancer-derived exosomes, specifically those under hypoxic conditions, enhance migration and invasion [17]. Using the PC3 cell line, prostate cancer cells were grown to confluence for a wound healing assay. With comparable initial wound sizes, cells grown in the presence of cancer-derived exosomes, specifically exosomes derived in the setting of hypoxia, demonstrated significantly increased cell migration and wound healing, with near complete closure of the wound. Examination of the cellular membranes revealed that exosome application decreased membrane E-cadherin expression, which is important in cell-to-cell adhesion, suggesting a pathway of the increased motility and invasiveness. Franzen et al. similarly demonstrated a decrease in cellular E-cadherin expression with the application of cancer-derived exosomes [2].

Breast cancer cell lines of increasing malignant potential have been demonstrated to release exosomes with increasing potency in promoting cell migration and invasion [54]. Harris and colleagues isolated cancer-derived exosomes from MCF-7, MCF-7 transfected with GFP-Rab27b-expressing plasmids, and MDA-MB-231 breast cancer cell lines, with low, intermediate, and high metastatic potential, respectively. When applied to several wound closure assays, the exosomes derived from moderate- and high-metastatic potential cells induced the greatest degree of cell migration and wound closure, while the exosomes from low metastatic potential cells demonstrated only a mildly increased effect compared to control. These exosomes were found to have increased protein expression of EMT promoters, including HSP90 and vimentin [55,56]. Similarly, lung cancer-derived exosomes from highly metastatic cells have also been found to increase vimentin and N-cadherin expression and decrease E-cadherin and ZO-1 expression when applied to epithelial cells, as well as increase motility and invasiveness [57]. Comparing lung-cancer derived exosomes from highly metastatic and non-highly metastatic lines, relative mRNA expression of mesenchymal markers was found to be significantly greater in the highly metastatic cancer-derived exosomes. Hsu demonstrated similar results with hypoxia-induced lung cancer-derived exosomes, which decreased cellular expression of tight junction protein ZO-1 in response to exosomal *miR-23a*, increasing permeability and transendothelial migration of HUVEC cells [46].

In breast and colon cancer, cancer-derived exosomes containing amphiregulin, an epidermal growth factor receptor ligand, have been implicated in cellular signaling and increased invasiveness [58,59]. Higginbotham et al. found that colon-cancer-derived exosomes contained several epidermal growth factor receptor ligands, including amphiregulin. Exosomes with amphiregulin were applied to either epithelial cells or breast cancer cells and demonstrated a several-fold increase in invasion on in vitro matrigel invasion assay.

Degradation of the extracellular matrix is another mechanism by which tumor cell migration and invasion is enhanced. Atay and coworkers elucidated the role of exosomes in this process in their work in gastrointestinal stromal tumors (GIST), in which cancer-derived exosomes alter nearby stromal cells to affect the tumor microenvironment [60]. In this study, oncogenic protein tyrosine kinase (KIT) was found to be packaged in cancer-derived exosomes. Upon co-culture of cancer-derived exosomes with a human uterine leiomyomatous smooth-muscle cell line, exosomes were taken up and cytosolic and membranous KIT expression was detected in 100% of observed cells within 24 h. Exposure to cancer-derived exosomes significantly increased cellular vimentin mRNA and protein expression. More impressive was the downstream KIT activity demonstrated in the previously KIT-naïve cells. Ultimately, upregulation of matrix metalloproteinase-1 was induced in the human uterine leiomyomatous smooth-muscle cells, which permitted an increased number of invasive GIST cells upon co-culture compared to cells without exosome exposure.

Finally, exosomes have been shown to transfer miRNA from an aggressive oral cancer cell line to induce cell motility and growth in the work by Sakha and colleagues [61]. Following recipient cell uptake of cancer-derived exosomes (from a highly metastatic oral cancer cell line, HOC313-LM), low malignant potential oral cancer cell lines demonstrate rapid proliferation compared to the non-exosome treated control group. When studied in a Transwell migration assay, exosome-treated recipient cells, compared to controls, demonstrated increased invasion. Analysis of cancer-derived exosomal miRNA content demonstrated upregulation of several miRNA with known oncogenic potential. When low malignant potential oral cancer cells lines were incubated directly with cancer-derived exosomes, or in a system with a 1 μm membrane to separate cell lines (thus allowing only transfer of acellular material including exosomes), an increase in miRNA expression was demonstrated on qRT-PCR.

As with the multiple examples above, it is clear that cancer-derived exosomes promote increasing cellular aggressiveness, with escalation of cellular motility, migration, and invasion. It has also been postulated that exosomes may function to concentrate proteins or RNA for signaling and transformation of nearby cells, as an explanation for the “field effect” phenomenon seen in several malignancies [59,62]. When specifically investigated, cancer-derived exosomes are demonstrated to not only carry, but also induce EMT with expression of mesenchymal markers in recipient cells, contributing to the progression to a more aggressive phenotype.

## 7. Exosomal Establishment of Pre-Metastatic Niche and Promotion of Metastasis

The idea that intercellular signaling is critical for the development of metastatic disease in the form of a pre-metastatic niche, as well as an organ-specific tropism of metastasis dependent on cancer type, is well established [19]. Recent development in the investigation of cancer-derived exosomes has established them as a key component of this process. Park and colleagues performed a functional and ontologic analysis of cancer-derived exosomal proteins and determined that the molecular and biological processes that occurred in the highest proportion were those associated with metastasis including cellular adhesion and extracellular matrix-receptor interaction [35].

Experimentally, this process has been eloquently described in the work of Costa-Silva and colleagues [63]. In their study, mice were treated first with injections of pancreatic ductal adenocarcinoma-derived exosomes from cell line PAN02, a cell line known for metastasis to the liver. Following three weeks of exosomal treatment, both treatment and control mice received an intrasplenic injection of PAN02 cells. At 21 days following intrasplenic injection, an increase in the incidence in liver metastasis was seen in mice treated with cancer-derived exosomes compared to PBS or normal pancreas-derived exosomal controls. To determine what cells took up the cancer-derived exosomes to promote metastasis, fluorescently labeled exosomes were then injected and liver cells examined by flow cytometry. Cancer-derived exosomes were preferentially taken up by Kupffer cells as opposed to other hepatocytes. Further, labeled normal pancreas-derived exosomes were not incorporated preferentially into any particular cell type. Finally, the authors elucidated that cancer-derived exosomal stimulation of Kupffer cells greatly upregulated TGFβ expression, a well-established inducer of EMT, resulting in increased fibronectin expression by hepatic stellate cells and ultimately development of the liver metastatic niche in this model [64]. This work indicates that cancer-derived exosomes are able to target a specific recipient cell type, as well as promote the development of metastatic disease in the target organ. 

Grange and coworkers demonstrated a similar process with cancer-derived exosomes isolated from a renal cell carcinoma line expressing mesenchymal stem cell marker CD105 [65]. When cancer-derived exosomes were injected intravenously into severe combined immunodeficiency (SCID) mice for five days preceding injection of renal tumor cells, the incidence of lung metastasis was significantly greater compared to vehicle or CD105^−^ expressing tumor cell controls. Analysis of the exosomes revealed upregulation of miRNAs associated with angiogenesis. In vitro application of the CD105^+^ cancer-derived exosomes stimulated HUVEC to organize into capillary-like structures on Matrigel, while also increasing the invasiveness on Transwell assays. 

In breast cancer miRNA *miR-105*, which is expressed and secreted by metastatic breast cancer cells, has been shown to be transferred by cancer-derived exosomes and to be an important factor in the development of metastasis [66]. In this study, mice were treated in vitro with cancer-derived exosomes isolated from breast cancer cell lines expressing high levels of *miR-105.* Following this pretreatment, mice were injected with breast cancer cells; subsequently, levels of lung and brain metastases were significantly increased compared to PBS and low *miR-105* exosomal controls. The mechanism for this interaction was explored, and exosomal delivery of *miR-105* to recipient cells was shown to downregulate tight-junction protein ZO-1 expression, resulting in increased permeability as measured in vitro. When cancer-derived exosomes were applied to human microvascular epithelial cells in vitro, again an increase in cellular migration as well as invasion in Transwell assay was demonstrated. It is interesting that, with the application of anti-*miR-105,* in vitro ZO-1 expression was increased and migration suppressed, while in vivo a more aggressive phenotype was inhibited, with decreased primary tumor volume and suppressed distant metastases.

Finally, Liu and colleagues detailed an investigation into the interaction between cancer-derived exosomes and Toll-like receptor 3 (TLR) in establishing a pre-metastatic niche in lung cancer [67]. It has been described that the stimulation of Toll-like receptors can be associated with multiple tumor processes, including growth and metastasis [68]. In their initial experiments, Liu et al. demonstrate that, in Tlr3^−/−^ mice, there was a significant reduction in spontaneous metastasis compared to wild-type mice. Further, when wild-type mice were injected with tumor-derived exosomes, there was an upregulation of TLR expression via an NF-κB- and MAPK-mediated pathways, an increase of mesenchymal markers (including fibronectin), as well as an increase in resultant metastasis. The NF-κB pathway has been demonstrated to be essential to the induction and maintenance of EMT, while the MAPK pathway appears to involve phosphorylation of ERK, JNK, or p38 MAPK, all of which occurred with the application of cancer-derived exosomes as above [67,69,70]. In contrast, when Tlr3^−/−^ mice were injected with cancer-derived exosomes, the level of spontaneous metastasis was comparable to negative controls. Investigation of this interaction between TLR3 and exosomes demonstrated that, when both are present, an upregulation of cytokines is apparent; however, without exosomes, or in the setting of silencing of TLR3, the upregulation in mesenchymal markers, and ultimately metastasis, does not occur. 

## 8. Conclusions

**Figure 2:**
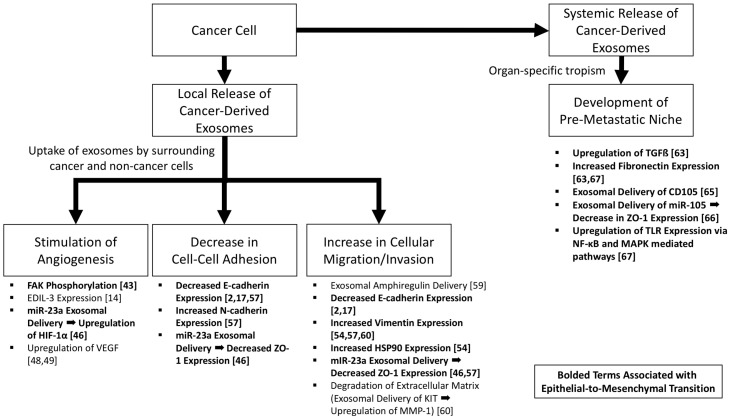
The role of cancer-derived exosomes in increasing tumorigenicity and epithelial-to-mesenchymal transition. Pathways associated with epithelial-to-mesenchymal transition are bolded.

Epithelial-to-mesenchymal transition represents a key process in which cancer cells develop a more aggressive, motile, less adhesive, and invasive phenotype. Cancer-derived exosomes represent methods of both local and distant intercellular signaling (Figure 2). Cancer-derived exosomes induce tumor cells and normal epithelial cells to acquire a more aggressive phenotype, with increased angiogenesis, disruption of tight junctions, increased motility, increased mesenchymal markers, and, in the setting of malignancies in which organs are in contact with a lumen (e.g., bladder and colon), may represent the explanation for the “field effect” that is seen clinically. Distantly, exosomes demonstrate preferential targeting of recipient cell types via surface proteins, explaining the tropism of metastasis inherent to specific cancer types, and in this setting establish a pre-metastatic niche which has been shown in several models [71].

The understanding of the role exosomes play in EMT remain in their infancy, and much study is needed. Directed studies into the effects of cancer-derived exosome application to target cells is needed to more fully understand the mechanisms behind migration, the loss in cell-to-cell adhesion, and the development of stem properties, which at this time are lacking. Further understanding of exosomal expression and intercellular signaling will improve the understanding of epithelial-to-mesenchymal transition as well as lead to novel diagnostic and therapeutic modalities targeting cancer progression.
